# Advancements in the treatment of Alzheimer's disease: a comprehensive review

**DOI:** 10.1590/1980-5764-DN-2024-0204

**Published:** 2025-04-07

**Authors:** Srushti Rajanna, Prajwal Pradeep Gundale, Apoorva Dev Mahadevaiah

**Affiliations:** 1 East West College of Pharmacy, Department of Pharmacy Practice, Bengaluru, Karnataka, India.

**Keywords:** Alzheimer Disease, Neuroprotective Agents, Neuroimaging, Genetic Therapy, Biomarkers, Doença de Alzheimer, Fármacos Neuroprotetores, Neuroimagem, Terapia Genética, Biomarcadores

## Abstract

Alzheimer's disease (AD) is a progressive neurodegenerative disorder characterized by cognitive decline, memory loss, and behavioral changes. Despite being the most common cause of dementia, effective treatments have been elusive. However, recent years have witnessed significant advancements in understanding and treating Alzheimer's. Key to these advancements is the shift toward targeted therapies tailored to individual genetic and biomarker profiles, promising more effective outcomes while minimizing side effects. The integration of advanced neuroimaging techniques has revolutionized early diagnosis and disease monitoring, enabling proactive intervention strategies that may alter disease trajectories. This review provides an overview of these advancements, focusing on disease-modifying therapies, symptomatic treatments, combination therapies, lifestyle interventions, biomarker development, innovative drug delivery systems, immunotherapy, gene therapy, and neuroprotective agents.

## RESUMO.

A doença de Alzheimer (DA) é um transtorno neurodegenerativo progressivo caracterizado por declínio cognitivo, perda de memória e mudanças comportamentais. Apesar de ser a causa mais comum de demência, tratamentos eficazes têm sido difíceis de encontrar. No entanto, nos últimos anos, houve avanços significativos na compreensão e no tratamento da DA. Um aspecto fundamental desses avanços é a mudança para terapias direcionadas, adaptadas aos perfis genéticos e biomarcadores individuais, prometendo resultados mais eficazes e ao mesmo tempo minimizando os efeitos colaterais. A integração de técnicas avançadas de neuroimagem revolucionou o diagnóstico precoce e o monitoramento da doença, possibilitando estratégias de intervenção proativas que podem alterar as trajetórias da doença. Esta revisão oferece uma visão geral desses avanços, com foco em terapias modificadoras da doença, tratamentos sintomáticos, terapias combinadas, intervenções no estilo de vida, desenvolvimento de biomarcadores, novas vias de administração de medicamentos, imunoterapia, terapia genética e agentes neuroprotetores.

## INTRODUCTION

**Figure 1:**
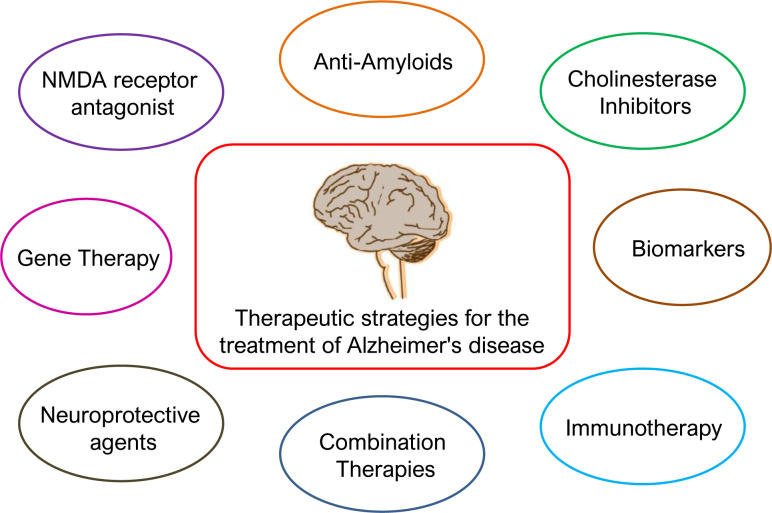
Clinical advancements in Alzheimer's treatment.

Alzheimer's disease (AD) affects millions of individuals worldwide, posing substantial emotional and economic burdens on patients, families, and healthcare systems^1^. Over 55 million people worldwide are living with dementia, with AD accounting for 60-70% of these cases. Each year, nearly 10 million new cases of dementia are reported, significantly impacting global health systems. In 2019, dementia incurred an estimated cost of $1.3 trillion to the global economy, with nearly 50% of these costs attributed to informal care provided by family and friends. Dementia is the seventh leading cause of death worldwide and a major cause of disability among older adults. Women are disproportionately affected, comprising 65% of dementia-related deaths and providing 70% of care hours for individuals with dementia^2,3^. Traditionally, treatments have been limited to managing symptoms without altering disease progression. Recent scientific progress has opened new avenues for therapeutic interventions aimed at modifying the disease course. This review synthesizes the latest research and clinical advancements in Alzheimer's treatment, highlighting their potential to transform patient outcomes (Figure 1).

The history of AD treatment is a convoluted narrative that intertwines medical breakthroughs, societal shifts, and persistent enigmas, each layer adding to the intricate tapestry of our understanding and management of this devastating condition^4^.

### Early observations and misunderstandings

The narrative of AD began in 1906 when Alois Alzheimer, a German psychiatrist and neuropathologist, first described the pathological hallmarks of the disease — amyloid plaques and neurofibrillary tangles — during an autopsy of a patient named Auguste Deter^5^. However, the implications of his findings were not immediately understood or appreciated. At the time, dementia was largely considered a normal aspect of aging rather than a distinct pathological entity^6^. This initial period was marked by a lack of targeted treatments, as the biomedical community grappled with the fundamental nature of the disease.

### The mid-20th century: emergence of neuroscientific insights

It was not until the mid-20th century that AD began to be recognized as a specific pathological condition rather than an inevitable consequence of aging. This period saw a burgeoning interest in neurobiology and the biochemical underpinnings of neurological diseases. Researchers started to explore the brain's biochemistry in greater detail, leading to early theories about the role of neurotransmitters, particularly acetylcholine, in cognitive function. However, therapeutic approaches were still rudimentary, primarily focused on symptomatic relief rather than addressing the underlying pathology^7,8^.

### 1980S: the cholinergic hypothesis and pharmacological advances

The 1980s marked a pivotal decade with the introduction of the cholinergic hypothesis, which posited that cognitive decline in AD was largely due to a deficit in acetylcholine, a neurotransmitter crucial for learning and memory. This hypothesis spurred the development of cholinesterase inhibitors, drugs designed to increase acetylcholine levels in the brain. Tacrine (Cognex), approved in 1993, was the first of these medications, followed by donepezil (Aricept), rivastigmine (Exelon), and galantamine (Razadyne). While these drugs provided modest symptomatic relief, the search for a cure remained elusive^5,9^.

### The amyloid cascade hypothesis and immunotherapy approaches

Parallel to the cholinergic hypothesis, the amyloid cascade hypothesis gained traction. This theory proposed that the accumulation of amyloid-beta plaques was the primary driver of Alzheimer's pathology. The late 1990s and early 2000s witnessed significant research efforts aimed at targeting amyloid-beta, including the development of immunotherapy strategies designed to clear amyloid plaques from the brain. Despite initial optimism, clinical trials for many amyloid-targeting therapies yielded disappointing results, casting doubt on the amyloid hypothesis and prompting researchers to reconsider alternative pathways^8^.

### Advancements in the treatment of Alzheimer's disease

#### Disease-modifying therapies

All currently approved disease-modifying therapies (DMTs) for AD consist of anti-amyloid monoclonal antibodies (mAbs). The approved drugs are aducanumab (Aduhelm^®^; Biogen, Cambridge, MA, USA) and lecanemab (Leqembi^®^; Eisai Inc. and Biogen, Cambridge, MA, USA). Aducanumab received accelerated approval based on evidence of significant β-amyloid (Aβ) plaque reduction observed on amyloid positron emission tomography (PET), likely indicating clinical benefit. Lecanemab initially obtaining accelerated approval following a phase II study and subsequent standard approval based on data from a phase III study. Donanemab, by Eli Lilly (Indianapolis, IN, USA) is currently being assessed for regular approval based on data from phase II and III clinical trials^10^.

While anti-amyloid mAbs share some similarities, they also exhibit distinguishing features. From a mechanism of action (MoA) perspective, all these therapies target high molecular weight fibrillar Aβ aggregates, resulting in significant Aβ reduction observed on amyloid PET scans and associated with amyloid-related imaging abnormalities (ARIA)^11^. The monoclonal antibodies vary in the amyloid species they target and in pharmacokinetic parameters like half-life, infusion frequency, and titration schedule^12,13^. Both studies included individuals with early AD, defined as mild cognitive impairment (MCI) due to AD and mild AD dementia. Notably, the definition of early AD varies across mAb trials, which could impact treatment selection for patients.

#### Aducanumab

Aducanumab (BIIB037; Aduhelm™) is a monoclonal auto-antibody (IgG1-mAb) that targets the N-terminal epitope made up of amino acids 3–7 of the Aβ42 peptide, showing greater affinity for fibrillar aggregates than monomers^14^. In June 2021, Aducanumab was granted accelerated approval by the U.S. Food and Drug Administration (FDA), becoming the first mAb targeting Aβ and the first DMT approved for AD; shortly after, it was also approved in the United Arab Emirates (UAE). The medication is recommended for early AD patients experiencing MCI or mild dementia who show signs of brain Aβ on amyloid PET or cerebrospinal fluid (CSF) tests^15^. Clinical trial data indicates that the optimal dosage for aducanumab is 10 mg/kg, administered through intravenous infusions every 4 weeks^15,16^.

#### Donanemab

Donanemab (Kisunla™) is a treatment that involves administering an anti-amyloid antibody via intravenous (IV) infusion every four weeks. The FDA has granted its traditional approval for its use in treating early AD, specifically for individuals with MCI or mild dementia caused by AD, with confirmed elevated beta-amyloid in the brain.

Donanemab can postpone the advancement of Alzheimer's in its early stages, granting individuals more time to engage in daily activities and maintain independence. Patients are encouraged to consult their healthcare provider to develop a personalized Alzheimer's treatment plan, considering the benefits and risks of all authorized medications and therapies.

#### Lecanemab

Lecanemab (Leqembi^®^) is a monoclonal antibody that targets amyloid-beta plaques. In contrast to aducanumab, lecanemab binds to protofibrils, which are a type of amyloid-beta known to be especially harmful to neurons. Clinical trials have demonstrated that lecanemab effectively decreases amyloid plaque buildup and decelerates cognitive decline in individuals with mild cognitive impairment or early AD. Ongoing research attempts to validate these results and further explore the therapeutic benefits of lecanemab. If successful, lecanemab could offer a viable alternative to aducanumab, providing patients and healthcare providers with another option for tackling AD^17^.

### Symptom management

#### Cholinesterase inhibitors

Cholinesterase inhibitors, including donepezil, rivastigmine, and galantamine, have been the cornerstone of symptomatic treatment for AD. These drugs work by inhibiting the enzyme acetylcholinesterase, which breaks down acetylcholine, a neurotransmitter essential for learning and memory. By increasing acetylcholine levels in the brain, cholinesterase inhibitors can help improve cognitive function and delay the worsening of symptoms in mild to moderate AD. Although they do not alter the underlying disease process, these medications provide meaningful symptomatic relief for many patients^18^.

#### NMDA receptor antagonists

Memantine, an NMDA receptor antagonist, is another key medication used to manage Alzheimer's symptoms. It works by regulating the activity of glutamate, another neurotransmitter involved in learning and memory. In AD, excessive glutamate activity can lead to neuronal damage and cognitive decline^10,19^. Memantine helps normalize glutamate activity, thereby protecting neurons and improving cognitive function. It is often prescribed in combination with cholinesterase inhibitors to enhance overall treatment efficacy.

### Combination therapies

The complexity of AD suggests that a multifaceted approach may be necessary for effective treatment. Researchers are increasingly exploring combination therapies that target different aspects of the disease. For instance, combining amyloid-targeting drugs like aducanumab with symptomatic treatments such as cholinesterase inhibitors and NMDA receptor antagonists may offer more comprehensive benefits. Additionally, combining drugs that target amyloid-beta with those that target tau, another protein that forms neurofibrillary tangles in AD, is an area of active investigation. The goal of combination therapies is to tackle multiple pathological processes simultaneously, potentially leading to greater improvements in cognitive function and slowing disease progression^20,21^.

### Lifestyle and non-pharmacological interventions

#### Cognitive training

Cognitive training programs involve structured mental exercises designed to improve specific cognitive functions such as memory, attention, and problem-solving. These programs can be delivered through computer-based exercises, group activities, or individualized therapy sessions. Studies have shown that regular cognitive training can help maintain or even improve cognitive abilities in individuals with AD. Moreover, cognitive training may enhance the brain's plasticity, helping it to adapt and compensate for neuronal damage^22^.

#### Diet and exercise

Diet and exercise play crucial roles in maintaining brain health and potentially reducing the risk of AD. The Mediterranean diet, rich in fruits, vegetables, whole grains, fish, and healthy fats, has been associated with a lower risk of cognitive decline and AD. Regular physical activity, including aerobic exercises like walking, swimming, and dancing, has been shown to improve cognitive function and reduce the risk of developing Alzheimer's. Exercise increases blood flow to the brain, promotes the release of neurotrophic factors that support neuron health, and helps reduce inflammation and oxidative stress. Social engagement and mental stimulation through hobbies and social activities also contribute to cognitive resilience and overall brain health^23^.

### Genetic and biomarker research

#### Genetic studies

Genetic research has identified several genes associated with an increased risk of AD. The APOE4 allele is the most well-known genetic risk factor, with individuals carrying one or two copies of this allele having a significantly higher risk of developing the disease. Understanding the genetic basis of Alzheimer's can lead to personalized medicine approaches, where treatments are tailored to an individual's genetic profile. This approach may improve treatment efficacy and reduce adverse effects^24,25^.

#### Biomarkers

Advancements in biomarkers have revolutionized the diagnosis and monitoring of AD. Imaging techniques, such as PET scans, can visualize amyloid-beta and tau deposits in the brain^18,19,26^. Fluid biomarkers, including CSF and blood tests, can measure levels of amyloid-beta, tau, and other proteins associated with AD. These biomarkers allow for earlier and more accurate diagnosis, as well as the monitoring of disease progression and response to treatment. Ongoing research aims to validate and improve these biomarkers, making them more widely available and less invasive^22^.

### Pathophysiological basis of Alzheimer's Disease biomarkers

The pathogenesis of AD is characterized by hallmark pathological features: extracellular amyloid-beta (Aβ) plaques, intracellular neurofibrillary tangles (NFTs) composed of hyperphosphorylated tau, and neuroinflammation. Biomarkers reflective of these processes serve as diagnostic and prognostic indicators.

#### Amyloid-Beta (Aβ) pathology biomarkers

CSF Aβ_42_/Aβ_40_ Ratio: The ratio of CSF Aβ_42_ to Aβ_40_ is a critical biomarker. A reduction in this ratio indicates Aβ plaque accumulation in the brain^27^. Studies using PET imaging with amyloid tracers, such as florbetapir, demonstrated that patients with low CSF Aβ_42_ levels had increased brain amyloid deposition, correlating with cognitive decline^28^.

Amyloid PET Imaging: PET tracers, such as 11C-PiB (Pittsburgh compound B), highlight amyloid deposition *in vivo*, aiding in differential diagnosis of AD from other dementias^29^.

#### Tau pathology biomarkers

CSF Total Tau (t-tau) and Phosphorylated Tau (p-tau): Elevated levels of t-tau indicate neuronal injury, while increased p-tau correlates with NFT pathology^30^. High CSF p-tau levels were linked to rapid cognitive deterioration, serving as a prognostic marker in MCI patients^31^.

Tau PET Imaging: Second-generation tau PET tracers, such as flortaucipir, are increasingly utilized to map the spread of tau pathology in AD patients^32^.

#### Neurodegeneration biomarkers

Neurofilament Light Chain (NfL): Elevated levels of NfL in CSF and blood are indicative of axonal damage, reflecting neurodegeneration in AD and other neurodegenerative diseases^33^.

MRI-Derived Atrophy Measures: Structural magnetic resonance imaging (MRI), detecting hippocampal atrophy, is a gold standard for assessing neurodegeneration^27^.

#### Neuroinflammation biomarkers

CSF and Plasma YKL-40: Increased YKL-40 levels, a marker of astrocytosis and microglial activation, are observed in AD^34^.

TSPO PET Imaging: PET imaging with tracers for translocator protein (TSPO) reflects microglial activation and neuroinflammation in the AD brain^35^.

#### Emerging blood-based biomarkers

Advancements in ultrasensitive detection technologies, such as single-molecule array (SIMOA), have enabled the development of blood-based biomarkers, offering a less invasive and cost-effective alternative to CSF biomarkers^36^.

Plasma Aβ_42_/Aβ_40_ Ratio: Reflects amyloid deposition with good correlation with amyloid PET findings^31^.

Plasma p-tau217 and p-tau181: Show promising diagnostic accuracy comparable to CSF and imaging biomarkers. Plasma p-tau217 demonstrated superior accuracy in distinguishing AD from non-AD dementias in a 2020 study^36^.

While biomarkers revolutionize AD management, challenges remain in accessibility, cost, and standardization. Emerging technologies, such as multiomics and artificial intelligence, promise to refine biomarker discovery and application. Multiomics approaches integrating proteomics, transcriptomics, and metabolomics are identifying novel biomarker candidates, such as lipids and metabolites involved in AD pathology^36^.

### Innovative drug delivery systems

Traditional drug delivery methods often face challenges in effectively delivering therapeutic agents to the brain due to the blood-brain barrier. Innovative drug delivery systems aim to overcome these challenges. Techniques such as nasal sprays can deliver drugs directly to the brain via the olfactory pathway, bypassing the blood-brain barrier. Focused ultrasound, combined with microbubbles^23^, can temporarily open the blood-brain barrier, allowing drugs to enter the brain more effectively. These advanced delivery methods have the potential to enhance the efficacy of Alzheimer's treatments by ensuring higher concentrations of therapeutic agents reach their targets in the brain^24^.

### Immunotherapy

#### Vaccines

Immunotherapy, including vaccines, is an emerging area of Alzheimer's research. Experimental vaccines aim to stimulate the immune system to recognize and clear amyloid-beta or tau proteins. Active immunotherapy involves injecting a patient with a modified form of the protein to elicit an immune response, while passive immunotherapy involves administering antibodies directly. Early trials of Alzheimer's vaccines have shown promise in reducing amyloid-beta levels^15^ and slowing cognitive decline. However, challenges such as ensuring the safety and efficacy of these vaccines need to be addressed before they can become widely available.

### Gene therapy

Gene therapy offers a potential strategy for addressing the genetic underpinnings of AD. Techniques like CRISPR/Cas9 allow for precise editing of genes associated with Alzheimer's, such as APOE4^26,37^. By modifying these genes, it may be possible to reduce the risk or severity of the disease. Gene therapy also holds potential for delivering therapeutic genes that encode proteins to protect neurons or enhance brain function. While still in the experimental stages, gene therapy represents a promising frontier in Alzheimer's treatment, with the potential to halt or even reverse disease progression^38^.

### Neuroprotective agents

Neuroprotective agents are compounds that protect neurons from damage and support their function. Research into these agents is ongoing, with several showing potential in preclinical studies^20,24,39^. For example, antioxidants can reduce oxidative stress, which contributes to neuronal damage in AD. Anti-inflammatory agents can mitigate neuroinflammation, another key pathological feature of Alzheimer's. Additionally, compounds that enhance mitochondrial function and energy production in neurons are being investigated. If proven effective, neuroprotective agents could complement other treatments by preserving neuronal health and function^40,41^.

A list of new drugs under clinical trials, developed to treat AD, are provided in Table 1 ^42–49^.

**Table 1:** List of new drugs under clinical trials, developed to treat Alzheimer's disease.

Serial No.	Drug	Therapeutic target	Safety	Side effects	Reference
1	Aducanumab	Amyloid-β (Aβ) plaques	Approved by FDA but later discontinued by its manufacturer (Biogen)	Amyloid-related imaging abnormalities (ARIA), headache	^42,43^
2	Lecanemab	Soluble Aβ protofibrils	Approved by FDA	ARIA, infusion-related reactions, headache	^42,44^
3	Donanemab	Amyloid β	Approved by FDA	ARIA, infusion reactions, brain swelling	^45^
4	Semaglutide	GLP-1 receptor	Ongoing trials; initial results promising	Nausea, vomiting, gastrointestinal issues	^42,46^
5	Simufilam	Filamin A (modifies tau and Aβ42 interaction)	Phase 3 trials underway; promising early results	Headache, fatigue, gastrointestinal issues	^42,43^
6	Galantamine	Cholinesterase inhibitor	Widely used; well-established safety profile	Nausea, vomiting, diarrhea, weight loss	^47^
7	Gantenerumab	Amyloid-β (Aβ) plaques	Ongoing clinical trials	ARIA, infusion-related reactions, headache	^42,48^
8	Solanezumab	Amyloid-β (Aβ) monomers	Ongoing clinical trials	Infusion-related reactions, ARIA	^42,43^
9	Bapineuzumab	Amyloid-β (Aβ) plaques	Discontinued due to safety concerns	ARIA, vasogenic edema	^42,45^
10	Memantine	NMDA receptor antagonist	FDA approved; well-established safety profile	Dizziness, headache, constipation	^42,43^
11	Troriluzole	Glutamate modulation	Phase 2/3 trials underway	Dizziness, headache, fatigue	^49^
12	Tideglusib	GSK-3 inhibitor (tau phosphorylation)	Phase 2 trials showed mixed results	Nausea, diarrhea, fatigue	^42^
13	LMTX	Tau aggregation inhibitor	Phase 3 trials underway	Gastrointestinal issues, fatigue	^43^
14	ANAVEX2-73	Sigma-1 receptor agonist	Phase 2/3 trials underway	Dizziness, headache, diarrhea	^42,46^
15	Blarcamesine	Sigma-1 receptor and muscarinic receptor	Ongoing clinical trials	Dizziness, headache, gastrointestinal issues	^42,44^
16	Elamipretide	Mitochondrial dysfunction	Ongoing clinical trials	Headache, nausea, fatigue	^44^
17	E2814	Tau propagation inhibitor	Ongoing clinical trials	Headache, fatigue, gastrointestinal issues	^42^
18	ALZ-801	Amyloid oligomers	Phase 3 trials underway	Nausea, headache, fatigue	^42,43^
19	Edaravone	Oxidative stress	FDA approved for ALS; trials for AD underway	Bruising, gait disturbance, headache	^42^

Abbreviations: FDA, Food and Drug Administration; ARIA, Amyloid-related imaging abnormalities; ALS, amyotrophic lateral sclerosis.

### Future perspectives and challenges in new drug development for the treatment of Alzheimer's disease

#### Future perspectives

##### Biomarker development

The advancement of biomarkers, particularly plasma biomarkers such as p-tau217 and amyloid-beta (Aβ_42_/_40_) ratios, offers promising tools for early and accurate diagnosis of AD^49^. These biomarkers could improve the identification of patients in the early stages, enabling timely intervention.

Advanced imaging techniques, including PET scans with new tracers for tau and amyloid, are likely to play a critical role in both diagnosis and monitoring disease progression.

#### Combination therapies

Future treatment strategies might involve a combination of drugs targeting different aspects of the disease, such as amyloid plaques, tau tangles, neuroinflammation, and mitochondrial dysfunction. This multifaceted approach could provide more comprehensive disease management.

#### Gene therapy and stem cell therapy

Gene therapy, aimed at correcting genetic mutations or modulating gene expression involved in AD pathology, represents a cutting-edge approach that holds potential for long-term solutions^50^.

Stem cell therapy could offer a way to replace lost neurons and restore function, although this area is still in the early stages of research.

#### Personalized medicine

Advances in genomics and proteomics could enable personalized medicine approaches, tailoring treatments based on an individual's genetic profile and disease biomarkers, potentially improving efficacy and reducing side effects.

### Challenges

#### Clinical trial design

Designing effective clinical trials for AD is challenging due to the disease's slow progression and variability in symptoms. Long trial durations and the need for large patient cohorts make these trials expensive and complex.

#### Regulatory hurdles

Gaining regulatory approval for new AD treatments can be difficult. The requirement for demonstrating significant clinical benefits in a disease with slow progression poses a significant hurdle.

#### Safety concerns

Many promising treatments have been associated with significant side effects, such as ARIA in amyloid-targeting therapies^51^. Balancing efficacy with safety remains a critical challenge.

#### Targeting amyloid-β

The amyloid cascade hypothesis has driven much of the therapeutic development for AD. Monoclonal antibodies such as aducanumab, lecanemab, and donanemab target amyloid-β plaques, which are hallmark pathological features of AD.

##### Aducanumab

Aducanumab showed plaque reduction in imaging studies. However, safety concerns, such as ARIA, including brain edema and microhemorrhages, were prominent. Phase III trials revealed ARIA-E in approximately 35% of patients, predominantly in ApoE4 carriers, necessitating rigorous MRI monitoring during treatment^16^.

##### Lecanemab

Similarly, lecanemab demonstrated efficacy in plaque reduction and modest cognitive improvement in the CLARITY-AD trial. However, its safety profile indicated ARIA in 21.5% of patients, with some cases resulting in discontinuation. Additionally, the risk of bleeding when combined with anticoagulants remains a critical concern for older populations^52,53^.

##### Donanemab

Results from the TRAILBLAZER-ALZ 2 trial suggest that donanemab can slow cognitive decline. Yet, ARIA rates were significant, affecting 37.9% of patients, with three treatment-related deaths attributed to complications of ARIA^10,54^.

These findings underscore the need for pre-treatment screening for genetic risk factors like ApoE4 status, and close safety monitoring during therapy^17^.

#### Tau protein inhibitors

Abnormal tau phosphorylation and aggregation are other key pathological drivers of AD. Tau-targeting therapies, such as semorinemab and gosuranemab, are in early-phase trials^55^.

##### Semorinemab

A Phase II trial indicated limited efficacy in slowing cognitive decline. Safety data highlighted relatively low incidences of severe adverse events compared to amyloid-targeting therapies, but long-term safety remains under investigation.

##### Gosuranemab

The TANGO trial was discontinued due to lack of efficacy. No significant safety signals were reported, but the modest adverse event rate suggests potential for further refinement^56^.

#### Small molecules and repurposed drugs

##### Blarcamesine (ANAVEX 2-73)

A sigma-1 receptor agonist with anti-inflammatory and neuroprotective properties, blarcamesine has shown promise in Phase II trials. Common adverse events included dizziness and fatigue, which were mild and transient.

##### Repurposed drugs

Drugs such as metformin, pioglitazone, and sildenafil have been investigated for their neuroprotective effects. While these agents are well-tolerated in non-AD populations, their impact on cognitive outcomes and safety in AD requires further exploration.

#### Gene therapies and RNA-based approaches

Emerging gene therapies and RNA-targeting treatments, including antisense oligonucleotides (ASOs) like tofersen, offer exciting possibilities for modulating disease progression. These approaches are still in early stages for AD, and safety concerns, such as off-target effects and immunogenicity, remain major challenges^57^.

Table 2 ^11,16,17,52–54,58–60^ describes the brief safety profile of new treatments in AD.

**Table 2:** Safety of new treatments in Alzheimer's disease.

Serial No.	Drug	Adverse effects	Key risk factors for complications	References
1	Aducanumab	ARIA-E (edema), ARIA-H (hemorrhages)	APOE ε4 genotype, high amyloid burden, superficial siderosis	^16^
2	Lecanemab	ARIA (edema and hemorrhages), headache	APOE ε4 genotype, microhemorrhages, anticoagulant use	^52,53^
3	Donanemab	ARIA (27% incidence), confusion	APOE ε4 genotype, advanced amyloid plaque burden, prior microhemorrhages	^54^
4	Semorinemab	Cognitive worsening, inflammation	Advanced tau pathology, older age	^11,17^
5	Gantenerumab	ARIA, nausea, infusion reactions	APOE ε4 genotype, comorbid vascular conditions, treatment dose	^58^
6	Verubecestat (BACE inhibitor)	Liver toxicity, cognitive decline	Dose-dependent effects, genetic predisposition, older age	^59^
7	ACI-35 (Anti-Tau vaccine)	Mild inflammation, behavioral changes	Interaction with other therapies, comorbidities (*e.g*., vascular issues)	^60^

Abbreviation: ARIA, Amyloid-related imaging abnormalities.

### Understanding disease mechanisms

Despite significant progress, there is still much to learn about the underlying mechanisms of AD. This incomplete understanding complicates the development of effective therapies^61^.

### Patient recruitment and retention

Recruiting and retaining patients for clinical trials is often difficult, especially in the early stages of the disease when individuals may not yet experience significant symptoms^62^.

### Cost and accessibility

Even when new treatments are developed, ensuring they are affordable and accessible to all patients remains a significant challenge. High costs of advanced therapies may limit their widespread use.

In conclusion, the landscape of AD treatment is rapidly evolving, with significant advancements across multiple fronts. Disease-modifying therapies, innovative drug delivery systems, genetic research, and lifestyle interventions collectively offer hope for altering the course of this devastating disease. Advancements in the treatment of AD are progressing on multiple fronts, including the development of novel biomarkers, combination therapies, gene and stem cell therapies, and personalized medicine approaches.

The future of AD treatment lies in a holistic approach that combines pharmacological interventions with lifestyle modifications, cognitive therapies, and supportive care. Integrating various treatment modalities could provide more comprehensive management of the disease. Early diagnosis through advanced biomarkers allows for early intervention, which is crucial for slowing disease progression and preserving cognitive function. This underscores the importance of routine screening and monitoring in at-risk populations. Emphasizing patient-centered care, where treatment plans are tailored to individual needs and preferences, will be vital. This approach can enhance treatment adherence and overall quality of life for AD patients and their caregivers. Ongoing research into the underlying mechanisms of AD, including neuroinflammation, synaptic dysfunction, and mitochondrial impairment, will be essential for developing new therapeutic targets and strategies.

However, significant challenges remain, including the complexities of clinical trial design, regulatory hurdles, safety concerns, an incomplete understanding of disease mechanisms, patient recruitment difficulties, and issues related to cost and accessibility. Addressing these challenges will be crucial for translating scientific discoveries into effective treatments that can improve the lives of patients with AD. By addressing these challenges and continuing to explore new scientific frontiers, the future of AD treatment holds promise for more effective, safe, and personalized therapeutic options. Continued research and clinical trials are essential to translate these advancements into effective treatments, ultimately improving the quality of life for Alzheimer's patients and their families.
